# Torsional locomotion

**DOI:** 10.1098/rspa.2014.0599

**Published:** 2014-11-08

**Authors:** D. Bigoni, F. Dal Corso, D. Misseroni, F. Bosi

**Affiliations:** DICAM, University of Trento, via Mesiano 77, 38123 Trento, Italy

**Keywords:** smooth contact, configurational force, material force, Eshelbian mechanics, motility

## Abstract

One edge of an elastic rod is inserted into a friction-less and fitting socket head, whereas the other edge is subjected to a torque, generating a uniform twisting moment. It is theoretically shown and experimentally proved that, although perfectly smooth, the constraint realizes an expulsive axial force on the elastic rod, which amount is independent of the shape of the socket head. The axial force explains why screwdrivers at high torque have the tendency to disengage from screw heads and demonstrates torsional locomotion along a perfectly smooth channel. This new type of locomotion finds direct evidence in the realization of a ‘torsional gun’, capable of transforming torque into propulsive force.

## 1. Introduction

Motion, based on self-propulsion or locomotion, is a research topic currently attracting strong attention in mechanics, robotics and biology. Since pioneering studies by Gray on serpentine propulsion [1–3], elastic bending of a rod has been shown to produce an axial tractive force, whereas torsion has never been linked to locomotion. In mechanics, torsion of elastic rods is an old, but still ongoing and important research topic [4–11], which is linked in this article to locomotion through the following model problem.

**Figure 1.**
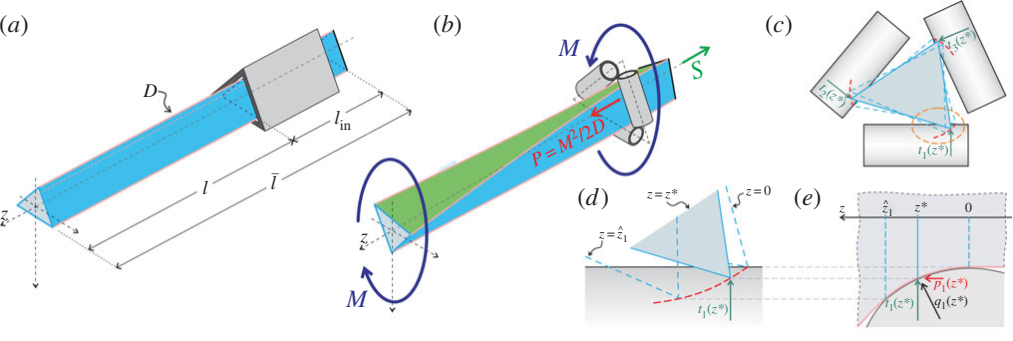
(*a*) Structural scheme of the elastic system employed to disclose the Eshelby-like propulsive force related to torsion; the cross section was sketched triangular, but can have any shape capable of resisting torsion. (*b*) Perturbative approach to analyse the Eshelby-like propulsive force *P* induced by the application of the torque *M*: the rod is imperfectly clamped to the sliding sleeve, in the sense that there is a misfit gap and the contact is idealized as with circular rollers. (*c*) Front view of the elastic rod, where the misfit gap is visible between cross section and torsional constraint. (*e*) The imperfect fitting of the rod/sliding sleeve system yields to contact over a certain line, so that the cross section ‘grasps’ the rollers along this line (sketched red in the details *c* and *d*), where the reaction *q*_*i*_(*z*), orthogonal to the profile, is acting. (Online version in colour.)

A rectilinear inextensible elastic rod is subjected to an applied torque at one end, whereas the other edge is inserted into a perfectly smooth and fitting female constraint, able to react to the applied moment (figure 1*a*). For instance, the elastic rod can be realized as a blade of thin rectangular cross section inserted in a flathead screw, or as a cylindrical rod of hexagonal cross section inserted in a hex socket. In these conditions, if *l* is the length of the rod between the application point of the torque *M* and the end of the female constraint, *D* the torsional rigidity (product of the elastic shear modulus *G* and the torsion constant *J*_*t*_) of the rod, the total potential energy of the system at equilibrium is

1.1 $$V(l)=-\frac{M^{2}l}{2D}.$$

Would the length *l* of the rod be fixed, nothing special follows, but, because this length is a free parameter, an ‘Eshelby-like’ or ‘configurational’ force^1^ *P* is obtained as negative of the derivative of the potential energy with respect to the configurational parameter, namely the length *l*

1.2 $$P=-\frac{dV(l)}{dl}=\frac{M^{2}}{2D},$$

parallel to the axis of the rod and expelling the rod from the constraint, if not balanced. This force, nonlinear in *M*, was never previously noted. It is, at a first glance, unexpected because of the smoothness of the female constraint, and simply explains why a screwdriver tends to disengage from a screw head. Even more interestingly, this axial force (1.2) can be understood as a propulsive force opening new possibilities for locomotion, while previously Lavrentiev & Lavrentiev [14] and Kuznetsov *et al.* [15] related locomotion of snakes and fish to the possibility of a system of releasing elastic flexural energy. The analytical expression, equation (1.2), for the propulsive force *P* is rederived and confirmed in §2 through two different methodologies, namely the variational and the perturbative approaches, whereas, in §3, the experimental evidence of this force is provided through its measure for different settings.

Torsional locomotion is finally proved in §4 through realization of a prototype, which generates a propulsive force from a release of torsional energy.

## 2. The existence of the torsionally induced axial force

The existence of the propulsive force *P*, equation (1.2), can be proved with a variational argument and recurring to a perturbation technique.

### (a) Variational approach

The total potential energy V of a rod, which can slide into a frictionless sleeve, subjected on the left end to an axial dead load *S* and on the right end to a torque *M* is (figure 1*b*)

2.1 $$V(\theta (z),\;l_{\mathrm{in}})=D\int_{l_{\mathrm{in}}}^{\bar{l}}\frac{{\left[\theta^{\prime }(z)\right]}^{2}}{2}\text{d}z-M\theta (\bar{l})-Sl_{\mathrm{in}},$$

where *z* is the coordinate along the rod's axis, *θ*(*z*) is the cross-section rotation in its plane, $\bar{l}$ is the total length of the rod and $l_{\mathrm{in}}=\bar{l}-l$ defines its portion lying inside the constraint, so that the kinematical boundary condition *θ*(*l*_in_)=0 and the statical boundary condition $\theta^{\prime }(\bar{l})=M/D$ follow.

Considering the rotation field *θ*(*z*) and the length *l*_in_ as the sum of the equilibrium configuration {*θ*_eq_(*z*);*l*_eq_} and the respective variations {*ϵθ*_var_(*z*);*ϵl*_var_} through a small parameter *ϵ*, the boundary conditions define as compatibility equations $\theta_{\mathrm{var}}(l_{\mathrm{eq}})=-\theta_{\mathrm{eq}}^{\prime }(l_{\mathrm{eq}})l_{\mathrm{var}}=0$ and $\theta_{\mathrm{var}}^{\prime }(l_{\mathrm{eq}})=-\theta_{\mathrm{eq}}^{\prime \prime }(l_{\mathrm{eq}})l_{\mathrm{var}}/2$, restricting the variations in the rotation field and in the length.

Equilibrium can be obtained by imposing the stationarity of the functional V to any small variation in the rotation field *θ*_var_(*z*) and in the length *l*_var_. The first variation $\delta_{\epsilon }V$ can be obtained as

2.2 $$\delta_{\epsilon }V=-\int_{l_{\mathrm{eq}}}^{\bar{l}}D\theta_{\mathrm{eq}}^{\prime \prime }(z)\theta_{\mathrm{var}}(z)\;\text{d}z+\left[D\frac{\theta_{\mathrm{eq}}^{\prime }{\left(l_{\mathrm{eq}}\right)}^{2}}{2}-S\right]l_{\mathrm{var}},$$

so that the equilibrium equations are

2.3 $$\begin{array}{rl} & \theta_{\mathrm{eq}}^{\prime \prime }(z)=0\;z\in [l_{\mathrm{eq}},\bar{l}]\\ \text{and}\; & D\frac{\theta_{\mathrm{eq}}^{\prime }{\left(l_{\mathrm{eq}}\right)}^{2}}{2}-S=0, \end{array}\}$$

the latter providing the axial equilibrium and showing the Eshelby-like or configurational force *P*, equation (1.2), once the former is solved taking into account the statical boundary condition $\theta^{\prime }(\bar{l})=M/D$.

### (b) Perturbative approach

The Eshelby-like force (1.2) can be obtained by introducing the assumption that the female constraint, though perfectly frictionless, has some geometrical imperfection. In particular, (i) there is a gap between the rod's cross section and the female, and (ii) the profile of the female is not sharply cut, but has a curvature (sketched for the sake of simplicity as circular in figure 1*b*,*e*). This imperfection will be shown to lead to the configurational force *P*=*M*^2^/2*D* (independently of the misfit gap and of the female's profile) and therefore to remain unchanged in the limit when the imperfection tends to zero (differently from the propulsive forces generated by bending [13]). This approach was introduced by Balabukh *et al.* [16] for a system subjected to bending, and is extended now to torsion where its results are complicated by the three-dimensional nature of the problem.

The elastic rod (with a polygonal cross section) of *z*-axis is assumed to be constrained by *N* (equal to 3 in figure 1) smooth cylindrical rigid profiles having a plane normal to their axes containing the *z*-axis. The shape of the cross-section boundary of each rigid profile (assumed circular for the sake of simplicity in figure 1*b*,*c*,*d*) is described by *g*_*i*_=*h*_*i*_(*z*), with *i*=1,…,*N*. The contact points may vary along *z*, so that the contact points are defined by the set C(z). Considering perfectly frictionless contact, at each contact point, a reaction orthogonal to the profile is acting (figure 1*c*,*d*,*e*), expressed by the line force *q*_*i*_(*z*), with i∈C(z), with transversal component *t*_*i*_(*z*) and axial component *p*_*i*_(*z*) given by

2.4 $$p_{i}(z)=t_{i}(z)h_{i}^{\prime }(z),$$

where a prime denotes a derivative with respect to *z*. The cross section of the elastic rod (triangular in figure 1), considered rigid in its plane, is subjected to an internal twisting moment *m*(*z*) varying along the elastic rod in the zone of contact and in equilibrium in its plane with the contact forces *t*_*i*_(*z*), so that the principle of virtual work written for an incremental torsion angle d*θ* and corresponding incremental displacements d*g*_*i*_=*h*_*i*_′(*z*)d*z* can be written as

2.5 $$\sum_{i\in C(z)}t_{i}(z)dg_{i}=m^{\prime }(z)d\theta ,$$

which, employing the constitutive equation d*θ*=*m*(*z*)/*Ddz* and the definition (2.4), becomes

2.6 $$\sum_{i\in C(z)}p_{i}(z)=\frac{{\left(m^{2}(z)\right)}^{\prime }}{2D}.$$

Therefore, a propulsive force *P* is generated that can be obtained as

2.7 $$P=\int_{0}^{\hat{z}}\sum_{i\in C(z)}p_{i}(z)dz,$$

where $\hat{z}$ is the point at which complete detachment from the rigid profiles occurs ($\hat{z}=\max_{i}\{{\hat{z}}_{i}\}$). A substitution of equation (2.6) into equation (2.7) and subsequent integration yields formula (1.2) for the propulsive force *P*, because *m*(0)=0, and $m(\hat{z})=M$ by equilibrium. Note that the thrust *P* is independent of the shape of the female's profile and of the amount of the initial gap, present between the rod and the smooth profiles, meaning that the amount of propulsive force, equation (1.2), is not affected by imperfections of the female constraints.

## 3. Experimental proof of the torsionally induced axial force

**Figure 2.**
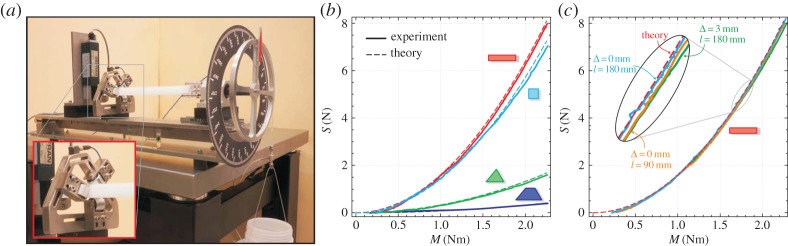
(*a*) The torsional apparatus working at imposed twisting moment *M*, with a detail of the realization of the frictionless sleeve to constrain a rod with triangular cross section. Torsionally induced axial thrust *S* measured as a function of the applied torque *M* and compared with theoretical predictions equation (1.2) for: (*b*) elastic rods differing in cross section and material (rectangular and square in PC, triangular and trapezoidal in HDPE) and (*c*) elastic rods in PC with rectangular cross section having different lengths (*l*={90;180} mm) and a null and a 3 mm misfit gap Δ. (Online version in colour.)

The system sketched in figure 1*b* has been realized to provide a direct experimental measure of the axial thrust *P*, equation (1.2). In particular, the torsional apparatus (figure 2*a*) has been designed and manufactured at the Instabilities Lab (http://ssmg.unitn.it/) of the University of Trento. The torque *M* is provided through a pulley (180 mm diameter) loaded at a constant rate with a simple hydraulic device in which water is poured into a container at 10 gr s^−1^ (the applied load is measured with a miniaturized cell from Leane, type XFTC301, R.C. 500 N). The elastic rod under twist is constrained against rotation by employing roller bearings from Misumi Europe (press-fit straight type, 20 mm diameter and 25 mm length), modified to reduce friction. Where the torque is applied, the elastic rod has been left free to slide axially through a double system, consisting of a linear bushing (LHGS 16-30 from Misumi Europe) mounted over a linear bearing (type easy rail SN22-80-500-610, from Rollon), so that longitudinal friction has been practically eliminated.^2^

Experimental results, presented in figure 2 for different cross section, length, elastic modulus and constraint condition of the elastic rod subjected to torsion, fully confirm the theoretical predictions. In particular, results obtained with rods of different lengths *l* and different misfit gaps Δ between the rod's cross section and the female constraint (*c*) show unequivocally the indifference of the Eshelby-like force from these parameters. Moreover, tests have been conducted with different elastic moduli for the rod employing high-density polyethylene (HDPE) and polycarbonate (PC) and different (thin rectangular, square, triangular and trapezoidal, corresponding to *D*={31.29;36.37;156.97;638.86} Nm^2^, respectively) cross sections (*b*). In all cases, the theoretical predictions have been found to be extremely close to experimental results (see the movie available as the electronic supplementary material for a sample of the test).

## 4. Torsional locomotion and torsional guns

Gray [1–3] has been the first to point out that a release of flexural elastic energy of a rod free of sliding in a frictionless channel can produce a locomotion force and Gray employed this force to explain fish and snake movement, so that a snake can propel itself producing bending by the backbone and its muscles. Within the terminology introduced in this article, the axial thrust produced during flexural deformation is the Eshelby-like force related to the release of elastic energy associated with curvature changes [17].^3^ It is therefore obvious to conclude that the configurational force *P*, equation (1.2), can be interpreted as a propulsive force capable of producing longitudinal motion through the application of a torque *M*.

**Figure 3.**
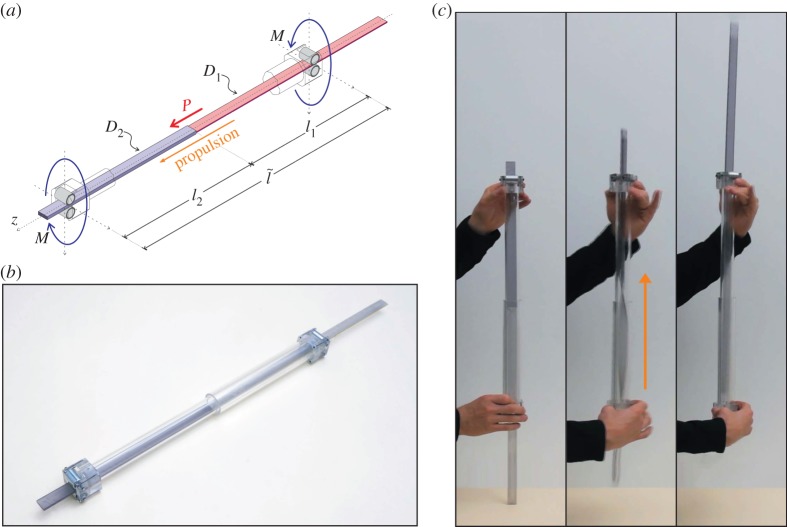
(*a*) Scheme of the model and (*b*) photo of the prototype of the torsional gun. An elastic strip made up of two laminae with different cross sections (so that one is ‘stiff’, *D*_2_=67.36 Nm^2^ and the other ‘soft’, *D*_1_=3.02 Nm^2^) is held between two pairs of roller bearings (at a distance $\tilde{l}=535\;\mathrm{mm}$). The system can be quickly twisted, so that a release of torsional elastic energy produces a propulsive force *P* that is enough to eject the elastic lamina. (*c*) The torsional gun in action: a sequence of three photos taken at 30 fps, showing that the propulsive force overcomes the gravity. (Online version in colour.)

To definitely prove that a torsional deformation can generate a longitudinal propulsion, a proof-of-concept device has been developed as shown in figure 3*a*,*b*. In particular, an elastic strip (19.5 mm wide and made in PC, weight 0.62 N) has been used, realized with two pieces with different rectangular cross section (one is 1.8 mm and the other 5.3 mm thick), so that one half of the strip, called ‘soft’ in the following, has *D*_1_=3.02 Nm^2^, whereas the other, called ‘stiff’, has *D*_2_=67.36 Nm^2^. The elastic strip is constrained with two pairs of roller bearings (at a distance $\tilde{l}=535\;\mathrm{mm}$) leaving the possibility of axial motion, but allowing the application of a torque *M* or a relative rotation *Θ*. Initially, the elastic strip is inserted within the rollers, so that the soft part of the strip has a length *l*_1_, and the stiff one has a length $l_{2}=\tilde{l}-l_{1}$. If a relative rotation *Θ* or a constant torque *M* is imposed between the two roller pairs, the total potential energy is, respectively,

4.1 $$V(\Theta ,l_{1})=\frac{D_{2}\Theta^{2}}{2(l_{1}(D_{2}/D_{1}-1)+\tilde{l})}\;\mathrm{and}\;V(M,l_{1})=-\frac{((D_{2}/D_{1}-1)l_{1}+\tilde{l})M^{2}}{2D_{2}},$$

so that the propulsive forces can be calculated as the negative of the derivative taken with respect to *l*_1_

4.2 $$P(\Theta ,l_{1})=\frac{D_{2}(D_{2}/D_{1}-1)\Theta^{2}}{2{\left(l_{1}(D_{2}/D_{1}-1)+\tilde{l}\right)}^{2}}\;\mathrm{and}\;P(M)=\frac{(D_{2}/D_{1}-1)M^{2}}{2D_{2}},$$

two formulae (the former holding for $l_{1}<\tilde{l}$) showing that the axial thrust is constant when *M* is imposed while it is a decreasing function of *l*_1_ when *Θ* is fixed. The elastic properties of the rod affect the amount of the propulsive force *P*. For instance, for a material with low shear modulus *G*, the torsional rigidities *D*_1_ and *D*_2_ of the projectile would decrease, whereas the propulsive force *P* would increase (decrease) for a given twisting moment *M* (for an imposed angle *Θ*).^4^ With the employed materials and geometrical set-up (*l*_1_=215 mm and *l*_2_=320 mm) and for an imposed angle *Θ*=*π*/2, the device realizes an initial propulsive force *P*=0.68 N, enough to overcome gravity when the device is held in a vertical configuration.

During manual use of the torsional gun, neither *Θ* nor *M* is precisely imposed, but a quick hand torsion of the device originates a propulsive longitudinal force able to eject the rod, even against gravity, see figure 3*c* and the movie available as the electronic supplementary material.

Note that, different from a bow or a catapult, in the ‘torsional gun’ the elastic deformation is stored in the projectile. The prototype of a torsional gun proves in an indisputable way that an axial motion can be produced via torsion, even in the absence of friction, so that a ‘flat animal’ can climb a frictionless narrow channel by employing a muscular torque.

## 5. Conclusion

Locomotion associated with torsional deformation of an elastic rod in a frictionless system has been introduced and substantiated both theoretically and experimentally, opening a new perspective in animal propulsion and in the mechanical design of deformable systems. The proof-of-principle ‘elastic gun’ shows how a torque can be transformed into a longitudinal thrust (or vice versa) without employing any mechanism, thus proving the realization of torsional locomotion.

## Footnotes

1 In the context of the virtual work principle, the Eshelby force is the generalized force associated with the axial kinematical (Lagrangean) variable, *P*−*S* in the following. The nomenclature ‘Eshelby-like’ force, used here to denote the propulsive force *P*, has been introduced by Bigoni *et al.*[12,13] for a different system subjected to bending. Results presented in this article confirm and extend their results to structures undergoing torsional deformation.

2 The Eshelby-like force has been measured using a Gefran OC-K2D-C3 (R.C. 50 N) load cell and all data have been acquired with a NI CompactDAQ system, interfaced with Labview v. 8.5.1 (National Instruments). The torsional device has been mounted on an optical table (from TMC, equipped with four Gimbal piston air isolators) to prevent spurious vibrations, which have been checked to remain negligible employing two IEPE accelerometers (PCB Piezotronics Inc., model 333B50).

3 Change in curvature is essential to produce energy release, so that (in the words of Gray [3]) ‘a snake cannot glide round the arc of a circle or along a perfectly straight line’.

4 The effects of a longitudinal extensibility of the rod could affect the propulsion process, although this aspect is not explored in this article (the rod is assumed inextensible). Furthermore, the propulsion could also be affected by buckling.
